# Editorial: Osteocytes in bone health and beyond

**DOI:** 10.3389/fcell.2025.1775689

**Published:** 2026-01-23

**Authors:** Lei Qin, Ye Chun Ruan, Da Jing, Francisca M. Acosta

**Affiliations:** 1 Department of Orthopedics, Shenzhen Nanshan People’s Hospital, The Affinited Nanshan Hospital of Shenzhen University, Shenzhen, China; 2 Department of Biomedical Engineering, The Hong Kong Polytechnic University, Kowloon, Hong Kong SAR, China; 3 Department of Biomedical Engineering, Air Force Military Medical University, Xi’an, China; 4 Division of Endocrinology, The University of Texas Health Science Center at San Antonio, San Antonio, United States

**Keywords:** bone health, osteoarthritis, osteoclast, osteocyte, osteoporosis, osteosarcoma

## Introduction

With the advancement of modern technologies, the mysterious mechanisms underlying bone—our body’s hardest tissue—have begun to be unveiled. Osteocytes, as the most abundant yet least well-studied cell type in bone, are attracting considerable attention due to their pivotal roles in maintaining bone health and systemic physiological homeostasis. Growing evidence demonstrates that osteocytes orchestrate bone formation and resorption, remodel the bone extracellular matrix, actively respond to physical stimuli, and function as endocrine cells to regulate distal organ physiology.

This Research Topic encompasses six original articles that collectively illustrate the functional roles of osteocytes in the local regulation of bone microstructure, intercellular communication, and various pathological contexts. In this editorial, we summarize these contributions, highlight their key research findings and translational implications, and outline future directions in osteocyte biology.

## Highlight from the Research Topic

Osteocyte death has long been linked to bone mass loss in various bone diseases. Zhao et al. summarized the mechanisms underlying different types of osteocyte death, including apoptosis, necrosis, necroptosis, ferroptosis, and pyroptosis. Despite their distinct regulatory mechanisms, various forms of osteocyte death promote osteoclast formation by either actively secreting pro-inflammatory and pro-osteoclastogenic cytokines—such as tumor necrosis factor alpha (TNF-α) and receptor activator of nuclear factor-kappa B ligand (RANKL)—or passively releasing pro-inflammatory damage-associated molecular patterns (DAMPs), such as high mobility group box 1 (HMGB1). Their work clarifies the potential mechanisms by which osteocyte death regulate osteoclast formation, aiming to improve the understanding of bone disease pathogenesis and identify potential therapeutic targets.

Sclerostin (SOST) is a Wnt signaling inhibitor primarily secreted by osteocytes that regulates bone formation and homeostasis. Wu et al. revealed that in the db/db mouse model of type 2 diabetes mellitus (T2DM), sclerostin expression was elevated and accumulated within the cortical osteocyte lacunar-canalicular system (LCS). This accumulation was correlated with increased expression of matrix-degrading enzymes—including Cathepsin K (CTSK) and matrix metalloproteinase 13 (MMP-13)—in osteocytes. Furthermore, *in vitro* experiments using recombinant sclerostin confirmed that the local effects of sclerostin within the LCS contribute to bone matrix degradation. This study elucidates the underlying mechanism of bone fragility in T2DM-related bone disease and highlights sclerostin’s local role during bone deterioration.

Osteosarcoma (OS) is the most common primary malignant bone tumor, in which the dynamic crosstalk between malignant cells and the unique bone microenvironment is mediated by intricate immune cell interactions. Zhang et al. analyzed the paradoxical roles of immune cell subsets in OS pathophysiology and systematically evaluated recent advances in immune cell-targeted therapeutic strategies for OS, providing valuable insights to optimize immunotherapeutic approaches in OS management.

Osteoarthritis (OA) is a major cause of lower limb mobility impairment in the elderly and the most prevalent joint disorder worldwide. Gao et al. summarized the current understanding of OA pathogenesis and symptom management. Jin et al. focused on the specific functions of E3 ubiquitin ligases (E3s) in OA pathogenesis: E3s influence OA progression by regulating the degradation of extracellular matrix proteins and inflammatory responses, thereby highlighting their roles and underlying mechanisms as potential targets for OA treatment. Another innovative OA therapeutic strategy is cartilage organoids, which are reported to target cartilage regeneration and structural repair. Gao et al. explored the development of cartilage organoids and discussed their future applications in drug screening and personalized therapies for OA.

Finally, Wu et al. reviewed the latest advances in osteocyte biology, focusing on their mechanotransduction via Piezo1 and integrins, regulation of osteoclastogenesis and osteogenesis, and crosstalk with the bone marrow microenvironment—including immune cells and vascular cells. Their systematic review highlights the accelerating pace of osteocyte-related research and the development of osteocyte-targeted therapeutic strategies for preventing osteoporosis, improving bone strength, and enhancing bone regeneration.

## Conclusion

Collectively, these contributions demonstrate how osteocytes regulate the local bone microenvironment, communicate with other cell types within the bone, and contribute to key bone pathological processes such as osteoporosis, OS, and OA. By integrating molecular, cellular, and systems-level knowledge, we highlight osteocytes as a pivotal therapeutic target for combating bone diseases and promoting bone regeneration. Looking ahead, further progress will depend on emerging approaches—including organoids therapy, CRISPR-mediated gene editing, and AI-driven multi-omics for precision medicine—which are accelerating osteocyte-related research and the development of targeted therapeutic strategies.

